# Lysine-Derived Maillard Reaction Products Inhibit the Growth of *Salmonella enterica* Serotype Typhimurium

**DOI:** 10.3390/pathogens12020215

**Published:** 2023-01-30

**Authors:** Catherine W. Y. Wong, Kaiwen Mu, David D. Kitts, Siyun Wang

**Affiliations:** Food, Nutrition and Health, University of British Columbia, 241-2205 East Mall, Vancouver, BC V6T 1Z4, Canada

**Keywords:** MRPs, *S. Typhimurium*, food safety, melanoidins, antimicrobial activity

## Abstract

An emerging consumer trend to purchase minimally heated and ready-to-eat food products may result in processing methods that do not effectively reduce pathogenic populations. Crude Maillard reaction products (MRPs) are naturally generated compounds that have been shown to display antimicrobial effects against pathogens. Crude MRPs were generated from reducing sugars (fructose (Fru), glucose (Glc), ribose (Rib) or xylose (Xyl)) with lysine and the melanoidin equivalence was measured using an absorbance of 420 nm (Ab_420_). The relative antimicrobial activity of each MRP was measured by examining both the length of lag phase and maximum growth rate. MRPs were found to significantly shorten the lag phase and decrease the maximum growth rate of *S. Typhimurium* (*p* < 0.05). Glucose-lysine MRP (GL MRP) was determined to have the highest relative melanoidin (1.690 ± 0.048 at Ab_420_) and its efficacy against *S. Typhimurium* populations was measured at 37 °C and at pH 7.0 and estimated on xylose lysine deoxycholate (XLD) agar. GL MRP significantly reduced *S. Typhimurium* populations by >1 log CFU/mL at 8 and 24 h after inoculation (*p* < 0.05). GL MRPs also further decreased *S. Typhimurium* populations significantly under thermal stress condition (55 °C) compared to optimal (37 °C) by ~1 log CFU/mL (*p* < 0.05). Overall, GL MRP demonstrated effective antimicrobial activity against *S. Typhimurium* at 37 °C and 55 °C.

## 1. Introduction

*Salmonella enterica* (*S. enterica*) is a facultative, Gram-negative bacteria that has been reported as one of the top pathogens causing foodborne illnesses in North America [1]. From 2017–2022, *S. enterica* has been implicated in numerous foodborne outbreaks in North America with foods such as chicken, ground beef, ground turkey, fish, frozen cooked shrimp, seafood, etc. [2]. *S. enterica* is resilient at a wide range of temperatures from −2 °C to 54 °C, with an optimum growth temperature at 37 °C and is able to survive in high acidic environments such as a pH of 2 when entering the stomach of a host [3]. A previous study indicated that *S. enterica* serovar Typhimurium displayed high acid resistance, with a survival level > 10% after being exposed for 2 h at pH 2.5 [4]. With the consideration that *S. enterica* is able to survive under severe conditions, controlling and minimizing *S. enterica* populations in the food system has been a major challenge in the past few decades.

An emerging consumer trend is to purchase minimally processed and ready-to-eat food products [5]. Minimally processed and ready-to-eat food products require less heat treatment. However, heat treatment is one of the most effective techniques to kill foodborne pathogens [6]. Consequently, issues have risen over the safety of minimally processed food products. A cooking method that has gained popularity in recent years is the sous vide cooking method. Sous vide cooking refers to having the food vacuum-sealed in heat-stable plastic pouches and then submerged in a water bath or a hot steam oven at temperatures lower than the minimum required cooking temperatures to kill pathogens presented by the U.S. Department of Agriculture Food Safety and Inspection Service and the Government of Canada [7,8,9]. Examples include heating red meat at 56 °C for less than 4 h and an average of 55 °C for fish and shellfish for sous vide cooking, while the recommended minimum cooking temperature is 62.8 °C for meat, fish and shellfish [7,8]. In 2014, there were three *S. enterica* Enteritidis illnesses in British Columbia, Canada, potentially related to consuming sous vide cooked foods [10]. From the three illnesses, two were potentially from sous vide style cooked eggs from one restaurant and the last was from sous vide cooked duck breast from another restaurant [10]. The investigation found the sous vide cooking method to be improperly carried out which amplified the *Salmonella* Enteritidis risk for illness in consumers [10].

There is currently no additional method to be used in concurrent with sous vide cooking temperatures to help reduce *S. enterica* populations. An important concern is if the initial *S. enterica* populations on the raw food ingredients are sufficiently high then mild sous vide cooking temperatures would be insufficient to decrease the populations to a safe level [11]. A proposed method of reducing *S. enterica* populations is the use of Maillard reaction products (MRPs) derived from foods thermally processed with time and temperature being factors that generate a complex network of compounds perceived important for improving the taste and appearance of foods. An additional important aspect of MRPs is the antimicrobial activity displayed by some; however, knowledge on specific conditions to elicit activity related to *S. enterica* is limited [12,13,14].

Previous studies have reported that the high molecular weight (HMW) compounds of MRP have relatively greater antimicrobial activity compared to lower or intermediate weight products [15,16]. One explanation for this finding is the potentially greater affinity of HMW products to exhibit metal-chelating activity which has been shown to result in cell membrane damage in Gram-negative bacteria [15,16,17]. Moreover, the efficacy of MRP to inhibit *S. enterica* growth under acidic and thermal stress conditions that are relevant to the food industry is currently unclear. Therefore, the objective of this study was to evaluate the effectiveness of crude, water-soluble MRP extracts generated from different reducing sugar-lysine model combinations for antimicrobial activity towards *S. enterica* Typhimurium under acidic and thermal stress conditions.

## 2. Materials and Methods

### 2.1. Preparation of Crude MRPs 

MRPs were conducted using reducing sugars (fructose (Fru), glucose (Glc), ribose (Rib) and xylose (Xyl)) (Sigma-Aldrich Co., St Louis, MO, USA) heated in the presence of lysine (Sigma-Aldrich Co., St Louis, MO, USA) at a ratio of 1:1 at 180 °C for 60 min in a Blue M convection oven (Blue Island, IL, USA) to generate the following crude MRP stock solutions: fructose-lysine (FL), glucose-lysine (GL), ribose-lysine (RL) and xylose-lysine (XL). The concentration of the crude MRP stock solutions were 500 mg/mL and the samples were subsequently collected and prepared by dissolving 0.4 g of crude MRP powder in 8 mL sterile deionized water. Samples were then homogenized and sonicated for 10 min in a FS-110 ultrasonic cleanser (Fisher Scientific, Hampton, NH, USA). Samples were then filtered through a 0.45 µm membrane filter (VWR International, Radnor, PA, USA) and stored at −20 °C for further chemical and microbiological experiments.

### 2.2. Determination of Crude MRP Stock Absorbance at 420 nm

A commonly used indicator to characterize the stages of Maillard reaction is the use of spectrophotometric measurements. At an absorbance of 420 nm (Ab_420_), brown pigments which are indicative of melanoidin equivalents are detected [18,19]. HMW MRPs, termed melanoidins for the purpose of this study, were measured in a 96-well plate at Ab_420_ at 23 °C using a microplate reader (SpectraMax M2; Molecular Devices, Sunnyvale, CA, USA).

### 2.3. Bacterial Strain and Growth Conditions

The bacterial strain used in this experiment was a *Salmonella enterica subsp. enterica* serotype Typhimurium FSL S5-536 (*S. Typhimurium*) from the ILSI *Salmonella* collection. The strain was maintained for long-term storage at −80 °C in tryptic soy broth (TSB, Becton, Dickinson and Company, Sparks, MD, USA) supplemented with 20% glycerol (VWR International, Radnor, PA, USA). Working stock of the strain was stored at 4 °C on brain heart infusion (BHI) agar (Becton, Dickinson and Company, Sparks, MD, USA) for a maximum of one month. An isolated colony of *S*. Typhimurium was inoculated into 5 mL of BHI broth (Becton, Dickinson and Company, Sparks, MD, USA) and the culture was incubated for 18 h at 37 °C with orbital shaking at 175 rpm. Approximately 10^9^ colony-forming units (CFU)/mL were obtained by diluting the *S*. Typhimurium culture with BHI broth until OD_600_ reached 0.8 ± 0.02. Subsequently, the *S*. Typhimurium culture was centrifuged at 1800 × *g* for 5 min at 20 °C using Microcentrifuge 5424R (Eppendorf, Harmburg, Germany) and washed twice with phosphate-buffered saline ((PBS); VWR International, Radnor, PA, USA) for the following steps in Section 2.4 and Section 2.5.

### 2.4. Growth of S. Typhimurium with MRP under Acidic and Thermal Stress Conditions

To assess the growth parameters (length of lag phase and maximum growth rate) of *S. Typhimurium* under acidic stress, pH 5.5 and control, pH 7.2 conditions were used. To reach pH 5.5, 1 M hydrochloric acid (VWR International, Radnor, PA, USA) was added to the BHI broth using an Accumet^TM^ AB 15+ Basic pH meter (Fisher Scientific, Hampton, NH, USA). One hundred and seventy µL of BHI broth with pH treatment (pH 5.5) and without pH treatment (pH 7.2) were dispensed into individual wells of a 96-well microtiter plate (VWR International, Radnor, PA, USA) with 20 µL of MRP stock solutions (FL, GL, RL and XL) from 2.1 and 20 µL of sterilized water (blank), respectively. The washed *S. Typhimurium* culture from Section 2.3 was then diluted to achieve a concentration of 10^4^ CFU/mL using buffered peptone water ((BPW); Becton, Dickinson and Company, Sparks, MD, USA) and 10 µL of the *S. Typhimurium* culture was added to each of the wells containing: (1) acidified BHI broth (pH 5.5) with MRP stock solutions, (2) acidified BHI broth (pH 5.5) without MRP stock solutions, (3) BHI broth (pH 7.2) with MRP stock solutions and (4) BHI broth (pH 7.2) without MRP stock solutions. The final concentrations of MRPs with *S. Typhimurium* were 0.5 mg/mL. The microtiter plate was then placed in a SpectraMax M2 microplate reader (Molecular Devices, Sunnyvale, CA, USA) for OD_600_ nm readings for 24 h with 10 min intervals at 37 °C.

Thermal resistance assay was performed as mentioned above, but 10 µL of the *S. Typhimurium* culture was added to each of the wells containing: (1) BHI broth (pH 7.2) with MRP stock solutions and (2) BHI broth (pH 7.2) without MRP stock solutions at 42 °C. Growth curves generated from the current study were fitted with the Gompertz model using the grofit package in R, version 4.1.1 (R, Inc., Boston, MA, USA) to estimate the length of lag phase (λ) and maximum growth rate (µ). Lag phase is the period of time in which the cells are adjusting to the environment before exponential growth [20]. Maximum growth rate is the highest rate of increase under favorable conditions for the cell [21].

### 2.5. Efficacy of GL MRP against S. Typhimurium 

A total of 5 mL of the *S. Typhimurium* culture from 2.3 was inoculated into 5 mL of BHI broth and 100 µL of GL MRP. A blank was used with 10 mL of BHI broth and 100 µL of sterile deionized water to ensure no cross-contamination occurred. All samples were incubated at 37 °C and the *S. Typhimurium* population was estimated at 0, 4, 8, 12 and 24 h after inoculation on xylose lysine deoxycholate ((XLD); Becton, Dickinson and Company, Sparks, MD, USA) agar. The agar was incubated at 37 °C for 24 h before retrieval for bacterial population estimation.

### 2.6. Growth of S. Typhimurium with GL MRP at 37 °C and 55 °C

Thermal resistance was performed 8 h after the inoculation of *S. Typhimurium* into 5 mL of BHI broth and 100 µL of GL MRP from 2.5. Eight h was chosen because the data from 2.5 indicated it was the end of the exponential phase. Ten µL of the *S. Typhimurium* inoculum from 2.5 was added to 990 mL PBS to reach 10^4^ CFU/mL. The diluted *S. Typhimurium* suspension was plated on the XLD agar prior to incubation as the negative control. The remaining diluted *S. Typhimurium* suspension was washed twice with PBS and the precipitated pellet was used for further experiments. To study the thermal resistance of *S. Typhimurium*, the pellet was dissolved in BHI broth and half of the samples were heated at 37 °C and the other half at 55 °C for 10 min. Afterwards, *S. Typhimurium* populations from the samples were estimated on XLD agar after incubation at 37 °C for 24 h.

### 2.7. Statistical Analysis

The melanoidin equivalence based on Ab_420_ from different sugar-lysine model systems from 2.2 was conducted with seven replicates and the absorbance was analyzed by one way analysis of variance (ANOVA), followed by Tukey’s Honest Significant Difference (Tukey’s HSD) for means separation. For 2.4, the experiment was similarly conducted with seven biological replicates and the length of lag phase and maximum growth rate of *S. Typhimurium* was analyzed by one-way ANOVA, followed by Tukey’s HSD for means separation. Three biological replicates were conducted for 2.5 and a one-way ANOVA was used to determine the effect of GL MRP on *S. Typhimurium* with Tukey’s HSD for means separation. Similarly, three biological replicates were conducted for 2.6 but the difference in *S. Typhimurium* populations with GL MRP at 37 °C compared to 55 °C was analyzed with a Student’s *t*-test. All samples were considered statistically significant with *p* values < 0.05. All statistical analyses were conducted with JMP, version 11 (JMP, Cary, NC, USA).

## 3. Results and Discussion

### 3.1. Sugar-Amino Acid Combinations to Generate HMW MRP Products

The melanoidin equivalent based on Ab_420_ from different sugar-lysine model systems is presented in Table 1. Different reducing sugars, such as Fru, Glu, Rib and Xyl, were reacted with lysine to generate crude MRPs (FL, GL, RL and XL) at 180 °C for 60 min. Of these four sugar–lysine combinations, it was the GL MRP that had the significantly highest melanoidin content (*p* < 0.05; Table 1). This is similar to a previous study that found model GL to contain a 45% and 15% higher melanoidin content compared to XL at pH 5.5 and 8.0, respectively [22].

### 3.2. Effect of MRPs on the Survival of S. Typhimurium under Sublethal Acidic and Thermal Stress Conditions

The two conditions tested in this study to evaluate the sensitivity of *S. Typhimurium* to different reaction models used to generate HMW MRPs included altering pH and temperature; both of which are commonly used to control foodborne pathogens in the food industry. Turbidity measurements (OD_600_) were used to assess cell density without differentiating between live or dead cells.

The lag phase of a bacterial growth cycle reflected the time it took for the bacteria to adjust to a new environment before it could undergo exponential growth [23]. All four MRP supplementations had no significant differences compared to control at pH 7.2 and at 37 °C (*p* > 0.05; Table 2). In contrast, in the acidic environment of pH 5.5, *S. Typhimurium* cells exposed to GL MRP had a shorter lag phase compared to other treatments (*p* < 0.05; Table 2). At 42 °C and at pH 7.2, samples with MRP treatment except for FL had a significantly decreased lag phase compared to controls (*p* < 0.05; Table 2). Thus, *S. Typhimurium* exhibited the fastest adaption to the acidic environment with GL MRP supplementation. When under thermal stress, *S. Typhimurium* exhibited the fastest adaption with GL, RL and XL MRP supplementations.

It is important to note that the composition of melanoidins from different sugar–lysine combinations may differ in respect to the physical, chemical or biological properties of pre-melanoidin components that form a complex mixture of HMW melanoidins [24]. These complex set of reactions define both intermediate (pre-melanoidin) and final (melanoidin) stages, that are sugar-specific in terms of final composition [19,24]. In addition to temperature, the pH is also a factor, as under slightly basic conditions, Schiff base and Amadori rearrangement, products will tend to lead to a 2,3-enolization reaction [24]. This is different if the conditions have an acidic pH, where the Amadori rearrangement is facilitated to a 1,2 enolization pathway [24]. Further dehydration and fragmentation results in numerous additional pre-melanoidin products formed during these intermediate stages, which collectively influence the final composition of MRPs and thus could contribute for the significant differences in antimicrobial activity and the length of the lag phase against *S. Typhimurium*.

Previous studies indicated that *rpoS*, a virulence factor, was known to support *S. enterica* survival in harsh environmental conditions and the expression of *rpoS* could be upregulated under acidic stress conditions [25,26,27]. Chalova et al. (2012) observed a 3.43-fold upregulation of *rpoS* expression when *S. Typhimurium* was exposed to MRP, which indicated the potential of MRP to modulate the stress response [28]. Recent studies indicated that deletion of *rpoS* in *S. Typhimurium* resulted in a longer lag phase when exposed to acidic stress conditions [25]. Overall, four MRPs in this study shortened the length of lag phase to different extents, compared to a control, which supports a previous study that demonstrated it was the variation on melanoidin content that contributed to different levels of antimicrobial activity [29].

All four sources of MRPs significantly decreased the maximum growth rate compared to the control at pH 5.5 and pH 7.2, at 37 °C, respectively, and also at pH 7.2 at 42 °C (*p* < 0.05; Table 3). However, the significant differences observed for MRP treatments derived from specific sugar–lysine combinations could not be distinguished despite the apparent difference in melanoidin content (Table 1 and Table 3). This result was surprising because different MRP sources can vary in component compositions, which could lead to unique differences in relative efficacies to inhibit the maximum growth rate of *S. Typhimurium*. The results showed that this was not the case with the MRP crude fractions recovered in this study. An important factor to note is the formation of hydrogen peroxide (H_2_O_2_) as a product of MRP synthesis [24,30]. H_2_O_2_ is a common disinfectant against pathogens such as *S. Typhimurium* and previous research has found H_2_O_2_ produced from MRP synthesis causing cell damage [30,31,32,33]. H_2_O_2_ was produced from all four sources of MRPs [24,30] and this would result in a common, non-specific-MRP effect on the maximum growth rate of *S. Typhimurium*. This could explain the insignificant effects noted between different MRP treatments, albeit significantly different from the control (*p* < 0.05; Table 3).

Hauser et al. (2014) showed that the concentration of MRP was an important factor towards an antimicrobial activity. The MRP used was recovered from 85% ethanol and at a reconstituted concentration of 16.1 g/L, was shown to completely inhibit *Escherichia coli* (*E. coli*) after 24 h [34]. An MRP concentration of 6.5 g/L had no effect on *E. coli* growth compared to control, after a 21-h treatment [34]. Rufian-Henares and De La Cueva (2009) reported similar findings where the highest concentration of melanoidin (8.0 mg/mL) inhibited the highest growth of *E. coli* ATCC 35150, followed by 5.0 mg/mL, 4.0 mg/mL, 2.5 mg/mL and 1.0 mg/mL [35]. From Table 1, although it was shown that GL MRP had the highest melanoidin content, based on an Ab_420_ index, the capacity to mitigate the maximum growth rate of *S*. Typhimurium was not significantly different from other sugar–lysine sources of MRPs (Table 3). A possible explanation for this finding is that the MRPs were relatively crude mixtures of HMW components [21] that, when combined, exceeded the threshold concentration to show differences in the maximum growth rate of *S. Typhimurium* between the different MRP treatments [19].

### 3.3. GL MRP Inhibits the Growth of S. Typhimurium at Optimal Conditions 

**Figure 1:**
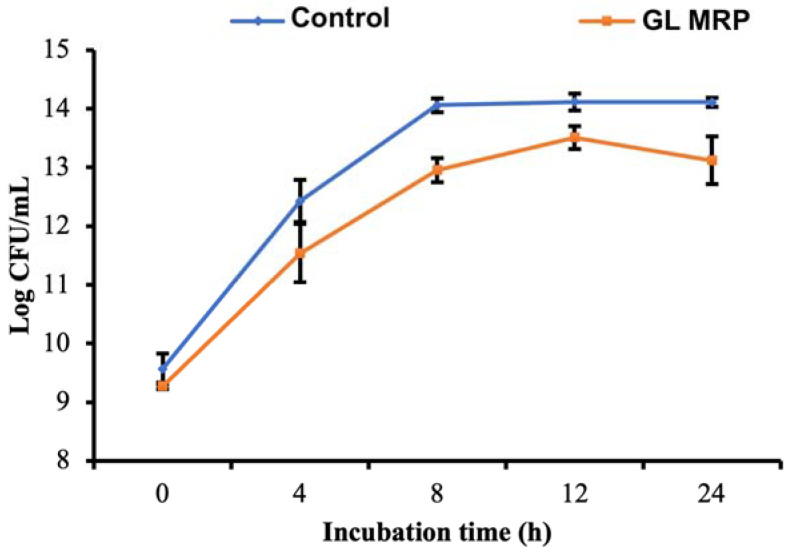
Growth of *S*. Typhimurium under no GL MRP supplementation (Control) and GL MRP supplementation at 37 °C on XLD Agar. Error bars represent standard deviation between three biological replicates.

In the present study, the GL model system was selected to generate MRPs, indexed by using Ab_420_ as an estimate of the highest melanoidin content (Table 1) in order to evaluate growth patterns of *S*. Typhimurium in a 24-h time frame at 37 °C. Figure 1 showed the growth of *S*. Typhimurium with GL MRP and without GL MRP supplementation (Control) on XLD agar. The GL MRP-treated *S*. Typhimurium populations were significantly reduced by ~1.5 log CFU/mL by 8 h and ~1 log CFU/mL by 12 h in comparison to the control after inoculation on XLD agar (*p* < 0.05; Figure 1).

The antimicrobial activity of HMW MRPs generated from reducing sugar–amino acid reactions have been studied previously with selected pathogens, and melanoidin content was identified as the antimicrobial component of MRPs [29,35,36,37,38]. Kukuminato et al. (2021) used melanoidin xylose-Phe and xylose-Pro against *Listeria monocytogenes* ATCC 1911 at 25 °C and found reductions of ~1 log CFU/mL with xylose-Pro and ~3 log CFU/mL with xylose-Phe after 12 h [29]. Another study used MRPs with glucose and fructose and found different concentrations of MRPs to have varying effects on *E. coli* and methicillin-resistant *Staphylococcus aureus* (MRSA) [38]. Glucose MRP reduced *E. coli* and MRSA by ~12% and 5% when using 3% glucose MRP, respectively [38]. The antimicrobial activity was increased when using 5% glucose MRP as *E. coli* and MRSA populations were reduced by ~45% and ~43%, respectively [38]. Fructose MRP was more effective against *E. coli* as 3% fructose MRP reduced *E. coli* and MRSA populations by ~17% and ~15%, respectively [38]. When fructose MRP concentrations were increased to 5%, *E. coli* populations were reduced by 60% but 5% fructose MRP was less effective against MRSA compared to 5% glucose MRP as populations were reduced by only 20% [37]. Bhattacharjee et al. (2021) found low concentrations of MRP (0.06X and 0.12X) could extend lag time by around 12 h and high concentrations of MRP (0.4X and 0.5X) were effective in decreasing populations of *Aggregatibacter actinomycetemcomitans* by up to 8 logs [37]. Ruffian-Henares and Morales (2008) found HMW MRPs exhibited a higher antimicrobial activity against *E. coli* compared to low molecular weight (LMW) MRPs [36].

Melanoidins are known to be negatively charged end-products of the Maillard reaction with metal-chelating activity for Mg^2+^, Fe^3+^ and Cu^2+^- ions [35]. Reducing cation availability, especially Fe^+^ on the outer membrane of Gram-negative bacteria, can initiate a sub-lethal injury, where irreversible changes in membrane function adversely affects nutrient transport to support growth [17,35,36]. One important example of an antimicrobial activity attributed to MRP -Fe^3+^ chelation concerns the potential interaction with siderophore-Fe^+3^ complexes [35]. The fact that *S. Typhimurium* is a Gram-negative bacterium is particularly important in this example, since Gram-negative bacteria secrete siderophores to bind Fe^3+^ [24]. Receptors and transporters located on the outer membrane of Gram-negative bacteria are required to utilize free Fe^3+^ [24]. The capacity of MRPs to chelate free Fe^+^ will interrupt Fe^3+^ binding to siderophores in Gram-negative bacteria, thus affecting cell membrane function for *S. Typhimurium* that results in reduced replication efficiency [24,35,39,40].

The Baranyi model for bacterial growth was selected over the Gompertz model due to better predictive capabilities that involve varying environmental conditions over time. There were four parameters that were assessed in the analyses: (1) maximum growth rate in CFU/mL/h (μmax); (2) initial cell density in CFU/mL (No); (3) maximum cell density in CFU/mL (Nmax) and (4) the difference of the maximum and minimum cell density in CFU/mL (ΔN). Based on the parameters obtained from the Baranyi model, all four parameters between control and GL MRP-treated *S. Typhimurium* cells were significantly lower compared to the control (*p* < 0.05; Table 4). These are compelling results to show a GL MRP-related antimicrobial effect against *S. Typhimurium*.

**Figure 2:**
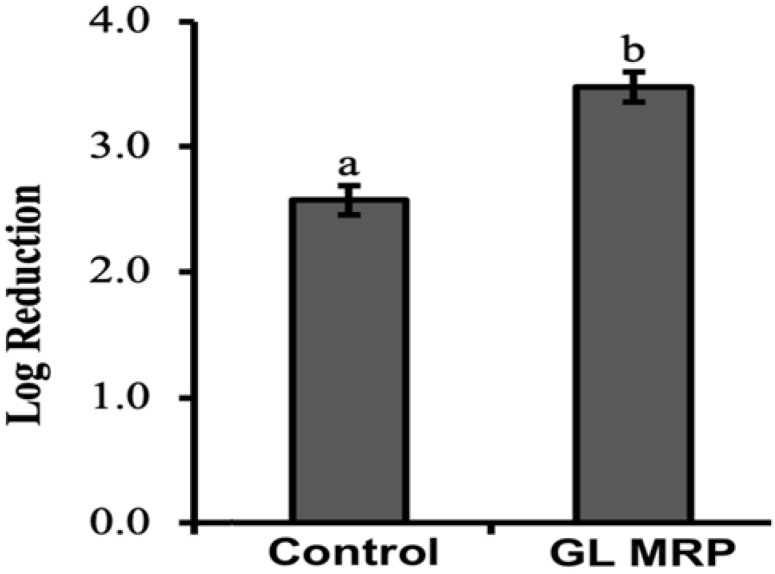
Log reduction in *S. Typhimurium* populations eight hours after inoculation without GL MRP supplementation (Control) and GL MRP supplementation heated at optimal condition (37 °C) and thermal condition (55 °C) for 10 min. Log reduction was obtained by calculating the difference in *S. Typhimurium* populations (log CFU/mL) on XLD agar between 55 °C and 37 °C. Means and standard deviations were calculated using data from three biological replicates. Different superscripts (a–b) indicate significant differences (*p* < 0.05) between treatments.

Figure 2 shows the log reduction in *S. Typhimurium* without GL MRP (Control) and with GL MRP treatment at optimal (37 °C) and thermal stress conditions (55 °C). Log reduction was obtained by calculating the difference in *S. Typhimurium* populations between 37 °C and 55 °C. Exposure of *S. Typhimurium* to GL MRP caused a significantly higher log reduction from 37 °C to 55 °C compared to the control, indicating that GL MRP accelerated the population decrease in *S. Typhimurium* at 55 °C (*p* < 0.05; Figure 2). Since high temperatures may accelerate molecular motion, the binding activity between GL MRP and Mg^2+^, Fe^3+^ and Cu^2+^ may be accelerated, which consequently aids in decreasing the population of *S. Typhimurium* [35]. The use of GL MRP against *S. Typhimurium* at 55 °C could be considered as a novel processing aid for foods heat-processed at lower temperatures. Recent food trends have been geared towards minimally processed foods to retain a high nutritional value and natural sensory qualities and a popular cooking method for this trend is sous vide cooking [8]. The temperature of sous vide cooking for certain food products such as fish, seafood and meat averages at ~55 °C and the food is maintained at this temperature for many hours or days [8]. However, the minimum recommended temperatures for cooking meat are to reach 62.8–71.1 °C and 62.8 °C for fish and shellfish internally [7,9]. If there is a sous vide cooking error and the fish, seafood or meat product is not heated for the entire duration of the time required, then foodborne pathogens may still be present and cause foodborne illness for the consumer. The surface application of GL MRP to foods that are cooked at temperatures lower than recommended temperatures could be considered as a potential aid to reduce foodborne illness risks in these food products.

## 4. Conclusions

The results of the current study have shown that the crude water-soluble MRPs have antimicrobial activity on *S. Typhimurium*. Due to the use of Ab_420_ as an estimation of melanoidin content, the compounds that form HMW MRPs could not be individually characterized. Future work should be completed to chemically characterize the HMW MRP components that contributed to the observed antimicrobial activity in order to determine the structure and functional activity of these compounds. From the MRPs used in this study, GL MRP was found to have the relatively highest melanoidin content and was able to reduce *S. Typhimurium* populations by >1 log CFU/mL after 8 and 24 h after inoculation. Therefore, the data support previous findings of an MRP antimicrobial activity. When *S. Typhimurium* was exposed to GL MRP, the difference in log populations on XLD of populations heated at 55 °C and 37 °C decreased by ~1 log CFU/mL compared to the non-GL MRP control. This supports the idea that GL MRP could be used as an effective aid when applied to the surface of a food product to reduce *S. Typhimurium* populations, when the temperatures used to process these foods are lower than the minimum-required cooking temperatures to kill foodborne pathogens. Future research on the efficacy of GL MRP against pathogens on foods heated at lower than required cooking temperatures is recommended for food products such as fish, seafood and meat at 55 °C; a common practice used for sous vide cooking. In addition, organoleptic testing will also need to be conducted on such products to test if distinct color and taste features considered important for seafood sensory quality are compromised in the presence of GL MRPs. 

## Figures and Tables

**Table 1:** Melanoidin equivalence (absorbance at 420 nm) of crude MRP (FL, GL, RL and XL) stock solutions heated at 180 °C for 60 min.

Crude MRP	Absorbance at 420 nm ^1,2^
FL	1.030 ± 0.006 ^b^
GL	1.690 ± 0.048 ^a^
RL	0.578 ± 0.011 ^d^
XL	0.813 ± 0.037 ^c^

^1^ All values are presented as mean ± standard deviation from seven replicates; ^2^ Values followed by different lower-case letters (a–d) indicate significant differences (*p* < 0.05).

**Table 2:** Length of lag phase λ (hours) of *S. Typhimurium* growth at pH 5.5 and pH 7.2 at 37 °C and pH 7.2 at 42 °C when incubated with MRP (FL, GL, RL and XL) treatments ^1^ for 24 h.

Crude MRP	λ at 37 °C		λ at 42 °C
	pH 5.5 ^2^	pH 7.2	pH 7.2 ^2^
Control (No MRP)	5.54 ± 0.11 ^a^	5.06 ± 0.05 ^a^	5.54 ± 0.29 ^a^
FL	5.23 ± 0.30 ^a^	4.70 ± 0.26 ^a^	4.95 ± 0.40 ^a,b^
GL	4.51 ± 0.31 ^b^	4.78 ± 0.29 ^a^	4.79 ± 0.26 ^b^
RL	5.18 ± 0.57 ^a^	4.59 ± 0.31 ^a^	4.83 ± 0.19 ^b^
XL	5.09 ± 0.33 ^a,b^	4.60 ± 0.28 ^a^	4.93 ± 0.45 ^b^

^1^ All values are presented as mean ± standard deviation from seven biological replicates. ^2^ Values within each column followed by different lower-case letters (a–b) indicate significant differences (*p* < 0.05).

**Table 3:** Maximum growth rate µ (/hour) of *S. Typhimurium* growth at pH 5.5 and pH 7.2 at 37 °C and pH 7.2 at 42 °C when incubated with MRP (FL, GL, RL and XL) treatments ^1^ for 24 h.

Crude MRP	µ at 37 °C		µ at 42 °C
	pH 5.5 ^2^	pH 7.2 ^2^	pH 7.2 ^2^
Control (No MRP)	0.128 ± 0.004 ^a^	0.230 ± 0.011 ^a^	0.163 ± 0.010 ^a^
FL	0.105 ± 0.005 ^b^	0.160 ± 0.042 ^b^	0.116 ± 0.046 ^b^
GL	0.087 ± 0.013 ^b^	0.160 ± 0.060 ^b^	0.097 ± 0.008 ^b^
RL	0.094 ± 0.017 ^b^	0.144 ± 0.032 ^b^	0.092 ± 0.004 ^b^
XL	0.095 ± 0.009 ^b^	0.127 ± 0.007 ^b^	0.101 ± 0.015 ^b^

^1^ All values are presented as mean ± standard deviation from seven biological replicates; ^2^ Values within each column followed by different superscripts (a–b) indicate significant differences (*p* < 0.05).

**Table 4:** The four parameters obtained from the Baranyi model for both control (No MRP) and GL MRP-treated *S. Typhimurium* on XLD Agar ^1^.

	µmax ^2^	No ^2^	Nmax ^2^	ΔN ^2^
Control	1.653 ± 0.059 ^a^	9.569 ± 0.261 ^a^	14.108 ± 0.084 ^a^	4.538 ± 0.205 ^a^
GL MRP	1.323 ± 0.251 ^b^	9.268 ± 0.068 ^b^	13.263 ± 0.125 ^b^	3.996 ± 0.132 ^b^

^1^ All values are presented as mean ± standard deviation with three biological replicates. ^2^ Values within each column followed by different lower-case letters (a–b) indicate significant differences (*p* < 0.05).

## Data Availability

Not applicable.
